# Interlog protein network: an evolutionary benchmark of protein interaction networks for the evaluation of clustering algorithms

**DOI:** 10.1186/s12859-015-0755-1

**Published:** 2015-10-05

**Authors:** Mohieddin Jafari, Mehdi Mirzaie, Mehdi Sadeghi

**Affiliations:** 1 Drug Design and Bioinformatics Unit, Medical Biotechnology Department, Biotechnology Research Center, Pasteur Institute of Iran, 69 Pasteur St, PO Box 13164, Tehran, Iran; 2 School of Biological Science, Institute for Research in Fundamental Sciences (IPM), Shahid Lavasani St, PO Box 19395-5746, Tehran, Iran; 3 Department of Computational Biology, Faculty of High Technologies, Tarbiat Modares University, Jalal Ale Ahmad Highway, PO Box 14115-111, Tehran, Iran; 4 National Institute of Genetic Engineering and Biotechnology (NIGEB), Pajoohesh Blvd, 17 Km Tehran-Karaj Highway, PO Box 161-14965, Tehran, Iran

**Keywords:** Protein-protein interaction network, Interlog protein network, Evolution, Network module

## Abstract

**Background:**

In the field of network science, exploring principal and crucial modules or communities is critical in the deduction of relationships and organization of complex networks. This approach expands an arena, and thus allows further study of biological functions in the field of network biology. As the clustering algorithms that are currently employed in finding modules have innate uncertainties, external and internal validations are necessary.

**Methods:**

Sequence and network structure alignment, has been used to define the Interlog Protein Network (IPN). This network is an evolutionarily conserved network with communal nodes and less false-positive links. In the current study, the IPN is employed as an evolution-based benchmark in the validation of the module finding methods. The clustering results of five algorithms; Markov Clustering (MCL), Restricted Neighborhood Search Clustering (RNSC), Cartographic Representation (CR), Laplacian Dynamics (LD) and Genetic Algorithm; to find communities in Protein-Protein Interaction networks (GAPPI) are assessed by IPN in four distinct Protein-Protein Interaction Networks (PPINs).

**Results:**

The MCL shows a more accurate algorithm based on this evolutionary benchmarking approach. Also, the biological relevance of proteins in the IPN modules generated by MCL is compatible with biological standard databases such as Gene Ontology, KEGG and Reactome.

**Conclusion:**

In this study, the IPN shows its potential for validation of clustering algorithms due to its biological logic and straightforward implementation.

**Electronic supplementary material:**

The online version of this article (doi:10.1186/s12859-015-0755-1) contains supplementary material, which is available to authorized users.

## Background

One of the important challenges in the interpretation of proteomic data is the detection of the cellular active process by exploring protein function. This newly emerging discipline; network science, has demonstrated that the majority of biological and evolutionary concepts make sense in the light of Systems Biology [1, 2]. Hence, the protein function, and consequently, cell function are more clearly demonstrated in the context of protein interaction network [3]. Protein-protein interactions make up the major branch in the study of protein interaction networks. From a biochemical view, these interactions can be divided into two categories: physical and functional [4, 5]. There are several methods employed to describe these interactions. The advantages and disadvantages of these methods have been widely reviewed [6–8]. Different limitations such as slow- and small-scale performances, inability to identify protein complexes, artificial interaction obtained from the in vitro assay and the operational restrictions, led to the discovery of methods that complement each other [9]. Some experimental methods are involved in determining physical and functional interactions [10, 11]. The phylogenetic profile, Rosetta stone, gene neighborhood and co-evolution are the most prevalent computational methods [11–13].

### Biological network modules

After constructing a Protein-Protein Interaction Network (PPIN), the next step is the exploration of the protein tasks within this complex circuit. As Alessandro Vespignani mentioned, “evolution thinks modular” [14]; a cell’s activity is a result of groups of interacting proteins, known as functional module (if they do not necessarily interact at the same time and place), in PPIN [15, 16]. Therefore, the PPIN modules should be identified and determined and then a biological function could be assigned to them based on the protein annotations. Sometimes this procedure is specifically more successful for the protein complexes that work together at the same time and place, rather than for the functional modules [6, 9].

Module detection can be divided into two approaches, namely graph clustering, and distance-based clustering. In the first approach, algorithms seek communities of the nodes in the graph that contain more intra-edges than inter-edges, e.g. Super Paramagnetic Clustering (SPC) [17], Highly Connected Subgraph (HCS) based on the Monte Carlo algorithm [18], Markov clustering (MCL) [19] and Restricted Neighborhood Search Clustering (RNSC) [20]. In the distance-based approach, the clustering algorithms e.g. the hierarchical or k-means are used so that the concept of distance and its associated measures in graph theory are applied as the similarity measures in the clustering. Some of the distances used in this approach are as follows: shortest path [21], number of edges [22, 23], shortest path profiles [24, 25] and a combination of distance and the statistical objects [26]. The detected modules are then well-characterized, biologically, based on information beyond the network topology such as gene expression, cell localization, virulence and knockout phenotypes [6, 27].

**Fig. 1:**
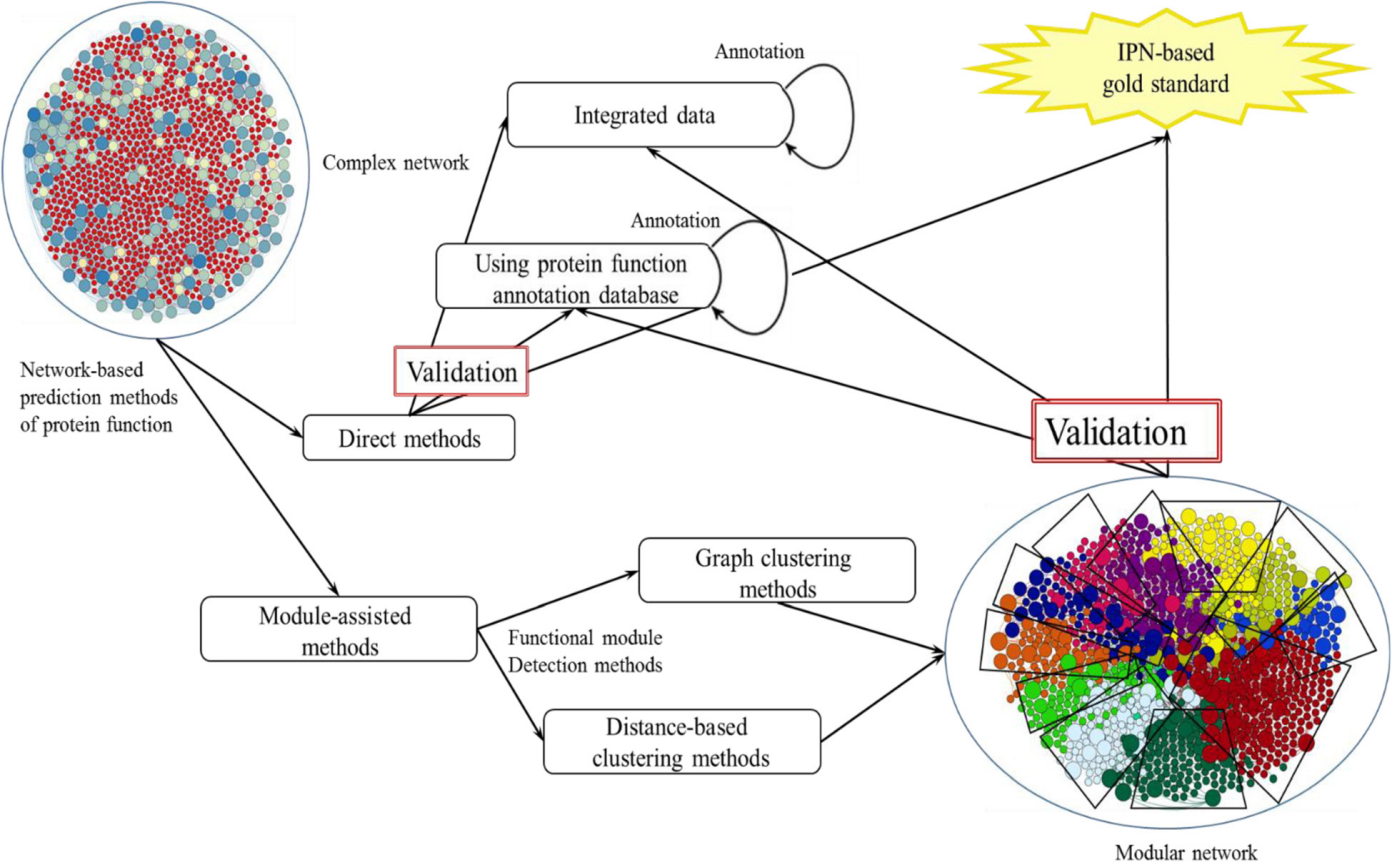
Function prediction procedures in network biology. Network-based prediction methods of protein function are described schematically. There are two approaches to explore function from network in biology, direct and module-assisted methods. In the direct method which is not our subject in this study, the annotation of gene/protein neighbors are used to predict function. But in the module-assisted methods, node’s community/neighborhood is principal for function prediction. These methods are also divided into two categories i.e. graph and distance-based clustering methods. The validation is the main step after finding modules in the PPIN or using direct methods. Any assignment of function based on annotation of neighbors or neighborhood should be evaluated by the different validating methods. We introduce the IPN-based validation for this purpose

The biological data are also applied in the next step for validation assays of module finding. The validation is based on the protein annotation homogeneity in the modules and these annotations could consist of functional, structural, local and/or interactomic information. Generally, the Gene Ontology (GO) and MIPS database are used in PPIN module validation [20, 28–32]. In addition, data from the gene expression profiles, co-localization and gene phenotype are also used [9]. It should be highlighted that the major presumption of the validation is that most of the proteins in a module, i.e. a statistically significant number of proteins, should be similar in intended attribute if the modules were identified correctly. A pictogram of this procedure is summarized in Fig. 1.

Although, protein complexes consist of functional and also physical interactions at the same time, it is obvious that many of the functional interactions are not in the data of the protein complexes. Several protein interactions happen transiently and indirectly, and as such are not detectable by empirical routine tests. These are important steps in the protein interactions which are lacking in the MIPS database [33–35]. In addition, modeling and representing the protein complex acquired by some experimental techniques, such as a graph (e.g. “spoke” and “matrix” model) is a challenging issue [36]. Furthermore, one cannot ignore the fact that the inherited experimental errors inherent to these problems and many more protein complexes, have not been fully studied as yet [37]. These flaws may lead to misinterpretation in the validation step if we use only the MIPS database.

On the other side, GO is a standard glossary of biological terms known as the first and most common reference for the Biological Process (BP), Molecular Function (MF) and Cellular Compartment (CC) of the proteins [38]. Additionally, a significant correlation between the node distances in some biological networks and the semantic similarity of their GO terms has also been reported [39–42]. Although the GO contains comprehensive and organized information, it has some limitations, namely, insufficient GO annotations (35–55 % false-negatives) [43], inaccurate GO annotation (false-positives, it should be observed that most of the annotations in GO are obtained by an indirect method such as gene manipulation as well as heterogeneous experimental and computational data), the functional diversity of proteins under different conditions resulting in different and sometimes conflicting annotations for one protein (false-positives) [44] and errors due to the manual annotation approach [45]. These deficiencies lead also to misinterpretation in the validation step.

### The goal of present study

Regarding the aforementioned restrictions in the validation step of module finding algorithms, we propose a network-based evolutionary benchmark as a complementary approach to solving some of the presented issues. Recently, in a companion study [46], we introduced a common network, that had low false-positives and tuned false-negatives. Using the four PPINs, a network with a high degree of conservation between four species was constructed. We call this common network the Interlog Protein Network (IPN) (Additional file 1). In the present study, the IPN, which is confirmed using experimentally proteomic data, has been suggested to be applied as a complementary benchmark in the validation of the different module finding algorithms, namely, Markov Clustering (MCL) [19], Restricted Neighborhood Search Clustering (RNSC) [20], Cartographic Representation (CR) [47], Laplacian Dynamics (LD) [48, 49] and Genetic Algorithm, to find communities in Protein-Protein Interaction networks (GAPPI) [16].

## Results and discussion

### Mitochondrial IPN

In the current study, the mitochondrial IPN of the four eukaryotic species was constructed. These species consisted of, human, rat, fruit fly and worm. The IPN was achieved through the interlog finding procedure of the mitochondrial PPIN of these species. In the other words, the IPN is an evolutionarily conserved network obtained from the overlap of orthologous proteins reinforced by gene expression. By pair-wise sequence alignment (≥30 %), the 226 Orthologous Protein Sets (OPSs) were obtained. Each OPS contained four orthologous proteins from the four species (83 human, 82 rat, 83 fruit fly and 80 worm). Finally, the IPN showed 29 nodes, 61 edges, 4.34° on average, a diameter of 6, and an average clustering coefficient of 0.625 (Additional files 1 and 2). This network represents the evolutionarily conserved topological network features shared among these species.

The expression data is used to empirically validate the IPN. The significantly high correlation between the protein concentrations endorsed the edges in the IPN. In fact, the correlation or co-expression network was reconstructed based on this concentration data and this network was compared to the IPN. In the previous study, the rat mitochondrial proteins (~500) were analyzed by several electrophoresis techniques [50]. It was claimed that different electrophoresis techniques are capable of fractionating proteins with different subcellular localizations [50]. Hence, the significant correlation between the expression profiles of the proteins in different electrophoresis implies they are co-localized proteins [51–53].

Of the total 563 proteins reviewed (UniProtKB database) 82 were in the mitochondria of rats which participated in OPSs. Later, 31 proteins were involved in the IPN and 20 of the 31 proteins were detected experimentally and involved in the co-expression network. In all, there were 23 significantly high correlations among these 20 proteins that matched with 13 of the 22 links in the IPN between these 20 proteins. The hypergeometric test confirms the reasonable matching ratio of the IPN ~60 % with respect to the edge match number in the rat PPIN 12 % with *p*-value 3.9 × 10–7 (Table 1).

**Table 1:** IPN expression data

Network name	STRING derived networks (Nodes and Edges)	Co-expression networks (Nodes and Edges)	Matched edges	Ratio of matched edges
Interlog protein network (IPN)	31, 63	20, 22	13	~60 %
Protein-protein interaction network (PPIN)	563, 2431	230, 1205	150	12 %

Using the expression proteomic data, a correlation network was constructed and compared to database derived networks i.e. IPN and PPIN. This evaluation was performed for rat mitochondrial proteins and related proteins and their links were considered. The number of nodes and edges in STRING derived (col 2) and co-expression (col 3) networks are presented in this table along with the number of matched or same edges in two corresponding networks (col 4). The edge matching ratios of these networks are represented (col 5)

### Comparison of network clustering methods

All the methods including RNSC, MCL, CR, LD and GAPPI were performed to discover modules in all four PPINs and the IPN. It should be noted that all of these algorithms are unsupervised and the network size affects the number of clusters. By defining the IPN as the benchmark, some external measures were used to validate all the methods. Next, some comparison indices were used, including Jaccard, Rand, Fowlkes-Mallows and Minkowski for all species. Except for the Minkowski index with the range [0, +∞) (where the values near to zero indicated the greater similarity) the other indices have a range [0,1] and the values closer to zero, indicate greater inconsistency.

**Fig. 2:**
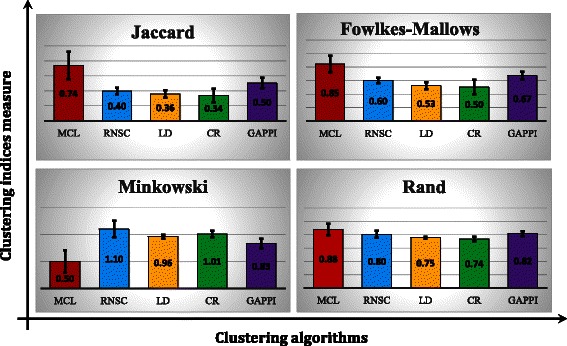
External clustering indices. The average of the calculated indices with SD (Standard Deviation) bars are shown graphically in the five clustering algorithms. As shown, the MCL outperformed in all the indices including even the Rand index with argued imperfection. Note that the range of the Minkowski index is [0, +∞) and the values (here is MCL) near to zero indicate the more similarity. But the other indices range is [0, 1] and the values near to zero specify the less similarity

The Rand index (unlike the Jaccard, Fowlkes-Mallows and Minkowski indices), measures the degree of similarity between two matrices as a function of the positive and negative agreements. Some studies claimed that the n_00_ value (Number of paired entities in the similarity matrices in which both are 0, see Methods) was often larger than the other values in most of the gene clustering studies and suggested using three other indices [54]. On the other hand, the Jaccard index is recommended due to its low variance [55]. However, the mean value of all the indices and standard deviations are shown graphically in Fig. 2 (All the values are shown in Additional file 2).

As represented in Fig. 2, our defined network showed that MCL outperforms the clustering methods of GAPPI, RNSC, LD and CR in terms of external measure indices. The range of the standard deviations showed that the MCL is more dependent on the size of the graph. This superiority was evident in all the indices, even in the Rand index with the above-mentioned imperfection. However, in the case of human PPIN as a large network, MCL and GAPPI cluster with similar accuracy (Additional file 2).

Meanwhile, the superiority of the MCL is compatible with the earlier results [32]. By distinct approach, they presented a comparative assessment of clustering algorithms and showed that the MCL was remarkably robust in graphing alterations and capable of the extraction of the complexes from the PPINs. CR took a long computation time and could not specify the modularity as well as the other algorithms within a reasonable time. The RNSC, LD and CR clustering showed similar ability to find the module robustly but the LD algorithm showed the lowest standard deviation among all the indices calculated. The GAPPI as the most recently proposed algorithm for this problem, works better than RNSC, LD and CR. This algorithm takes second place after MCL in all comparisons and it clusters large network same as MCL. This pattern was almost repeated in the recent study [16] using MIPS as gold standard.

### Biological evidence

The biological relevance of the proteins in each module detected by MCL was assessed. By using the Enrichr tool [56], three well-known biological standard databases are used namely; Gene Ontology (Biological Processes) [38], KEGG [57] and Reactome [58]. The result shows that each module enriched significantly and annotated separately (Additional file 3). Briefly, in 3 modules of this conservative IPN, the results are as follows. The first module is related to citrate/TCA cycle and oxidation phosphatase based on these ID numbers (GO:0006099, GO:0022904, GO:0022900, ko00020 and ko00190). The second module is related to Mitochondrial protein import based on these ID numbers (GO:0006626, GO:0070585, GO:0072655 and GO:0006839). The last module is related to Mitochondrial translation, ribosome and nucleoside biosynthetic process based on these ID numbers (GO:0046031, GO:0009133, GO:0046033, GO:0009135, GO:0009179, ko03010, ko00240 and ko00230). These results are compatible with our earlier study about biological meaningful communities in IPN as a pure evolutionary extract of mitochondrial PPIN [46].

## Conclusion

There are several module detection methods based on different approaches. Validation assays are required to compare and select the best one for network analysis. The major prerequisite for validation is the determination of the reliable benchmark. A standard topological and functional PPIN helps us to assess and verify the PPIN modularity results. In the earlier studies, researchers used the MIPS or GO dataset as the gold standard in validation assays. As mentioned earlier, these datasets are not point-device gold standard and each one has its own particular shortcomings. In other words, these databases have been designed with specific purpose and are diverse conceptually [59].

In the current study, we used the pair-wise sequence alignment and comparative interactomics of evolutionary distant species to reconstruct a conserved and common network that can be used as the benchmark or ground truth. The proposed benchmark does not have the above-mentioned limitations. First, the edges (interaction data) in IPN and associated compared networks are generally of the same origin. This implies that if the edges of the associated compared networks are predicted and designated computationally, this benchmark is also constituted from the computational data and so on for experimentally identified interaction. In other words, the IPN edges are a result of the filtering procedure (see Additional file 4) and they do not originate from logically distinct methods. Second, the IPN reconstruction procedure most likely leads to a network with low false-positives and tuned false-negatives. This issue has a high impact on the assessment results in the validation step. Third, the reconstruction of IPN is possible for all the sequenced proteins and genes that are well-conserved across multiple species with predicted interactions. It implies that this approach does not require special expensive and time consuming techniques to generate the experimental data and evaluate the molecular networks.

Similar to the previous result [32], but dissimilar in approach, we found MCL to be the outstanding algorithm based on its performance in the comparison study. In the traditional method, the MIPS database was used to evaluate the different clustering methods. The sensitivity and accuracy of the different methods was also examined by adding and subtracting the edges i.e. artificial false-positives and negatives (Note that their tests did not contain large size changes). Our findings about GAPPI implementation are also consistent with the prior study [16], which showed the improving ability of a genetic algorithm to search modules in PPINs based on the MIPS database.

However, interaction data was retrieved from the STRING database which includes different sources of information, including various experimental, computational and even text mining methods [60, 61]. In addition, an independent set of empirical data was applied and the IPN quality was experimentally confirmed. However, our goal was to search for and introduce a method that could segregate the functional modules. It should be noted again that a functional module means a group of cell components and their interactions that do or do not promise specific biological functions at the same time and place. So, these modules also include all the protein complexes. Therefore, the validation standard should not lack the functional interaction data. MCL is the superior module detection method in exploring the protein complexes and also for the functional modules based on the previous [32] and current studies, respectively. In addition, in terms of different graph sizes, it appears that MCL is not as robust as the other algorithms based on the range of the standard deviation.

In this study, we suggest the IPN to justify the modularity results of any PPIN due to three preponderances mentioned above. The graph clustering algorithm would be inefficient if it could not find the modules analogously in the individual PPINs and IPN as a purified, conserved and confirmed network. This approach to make a new benchmark may also help to assess and verify other biological networks e.g. gene regulatory networks or gene correlation networks and other biological network analysis methods such as network motif finding or orienting PPINs, which are subjects for further research. Again, this approach uses evolutionary concept i.e. conservedness to evaluate the biological networks. This is reminiscent of the well-known quote, “Nothing in biology makes sense, except in the light of evolution” [62].

## Methods

### Interlog protein network (IPN)

Construction of the common PPIN or IPN has been described earlier in detail [46]. Briefly, the mitochondrial reviewed proteins were retrieved from the four eukaryotic model species (Rattus norvegicus, Drosophila melanogaster, Caenorhabditis elegans and Homo sapiens) from the UniProtKB database (UniProt release 2013_02) [63]. Then, Using the Needleman and Wunsch algorithm, the homologous proteins were identified in the OPSs. In the next step, four distinct mitochondrial PPINs of the four species were identified from the STRING database (Ver. 9) [61]. The four PPINs were elicited with the default value in the database by all the prediction methods. Finally, by applying a stringent rule that is the existence of interlog in all four species, the mitochondrial IPN of these species was enucleated.

To explain more, an edge links two OPSs, say OPS1 = (p1h, p1r, p1f, p1w) and OPS2 = (p2h, p2r, p2f, p2w), if the protein pairs i.e. (p1h, p2h), (p1r, p2r), (p1f, p2f) and (p1w, p2w), have interaction based on STRING database (h, r, f and w indicate human, rat, fruit fly and worm respectively). Therefore, those OPSs which do not satisfy this condition will not be used in the IPN. In other word, the IPN was constructed in a way that each edge between two OPSs in the IPN indicates the six interlogs in these species (In this example, if it is a link between OPS1 and OPS2, then there are six interlogs i.e. ((p1h, p2h) and (p1f, p2f)), ((p1h, p2h) and (p1w, p2w)), ((p1h, p2h) and (p1r, p2r)), ((p1r, p2r) and (p1f, p2f)), ((p1r, p2r) and (p1w, p2w)) and ((p1f, p2f) and (p1w, p2w))). It should be noted that in each step some proteins are pretermitted to discern conserved structures (Additional file 4).

### Proteomics data

The results of the mitochondrial proteomic study of rat [50] were used for the empirical evaluation. In the shotgun proteomics strategy, the rat liver proteome with different cellular compartments was detected and quantified by several gel-based fractionation techniques. In the present study, normalized peptide counts were used to estimate the protein concentrations in a label-free quantification method. Then, Pearson correlation was applied to find the correlated proteins (|r coefficient| ≥ 0.7, *P*-value ≤ 0.05). According to the distinction made by the electrophoresis methods, the correlated proteins are likely co-localized. And, also as discussed earlier, co-localization can confirm the protein-protein interaction. Later, the ratio of the correlated proteins in the rat PPIN and IPN was computed separately and compared with the hypergeometric test (*P*-value ≤ 0.001). Thus, the IPN edges were examined by independent experimental data statistically.

### Network clustering algorithms

In the present study, five well-known different clustering algorithms (MCL, RNSC, CR, LD and GAPPI) were used to cluster the PPINs and IPN. The general characteristics of each algorithm are shown in the Table 2 and the associated references [19, 20, 47–49]. We presented further details regarding these algorithms in the Additional file 5. We clustered all the PPINs of species and IPN independently.

**Table 2:** Main features of the graph clustering methods presented in this study

	Markov clustering (MCL)	Restricted Neighborhood Search Clustering (RNSC)	Laplacian dynamics (LD)	Cartographic Representation (CR)	Genetic Algorithm to find communities in Protein-Protein Interaction networks (GAPPI)
Type	Flow simulation & Pagerank centrality	Cost-based local search	Multiscale modular structure	Inter- and intramodule connection	Search inspired by natural evolution
Allow multiple assignations	No	No	No	No	No
Allow unassigned nodes	No	No	No	No	No
Edge-weighted graphs supported	Yes	No	Yes	No	No
First application	Protein family detection	Protein complex prediction	High modularity partitions of large (more than million) networks finding	Metabolic network	Protein-protein interaction networks
Availability	http://rsat.scmbb.ulb.ac.be/rsat/index_neat.html	http://rsat.scmbb.ulb.ac.be/rsat/index_neat.html	Upon Gephi program	Upon request	http://staff.icar.cnr.it/pizzuti/codes.html
Developer (Year)	Enright A.J. et al. (2002) [19]	King A.D. et al. (2004) [20]	(1) Lambiotte R. et al. (2007) [49];	Guimera R. & Amaral LAN (2005) [47]	Pizzuti C. & Rombo S. E. (2014) [16]
			(2) Blondel V.D. et al. (2008) [48]		

### Evaluation of the clustering results

After clustering, validation is required to confirm the results or compare the different methods. A new benchmark was introduced, i.e., IPN in the validation step, so that the modules corresponding to each of the PPINs are compared with the IPN’s modules. In fact, the IPN was used as the ground truth in the standard external measures assay. Note that the clustering results on the PPIN are restricted to those proteins also in the IPN. It was expected that the successful algorithm should be able to find the modules analogously in PPIN and IPN. In order to assess the clustering results, the similarity matrices (Symmetric binary matrices) of clustering results were constructed, such that a 1 indicated placing two objects in the same cluster or module and a 0 indicated the opposite. Then, the entities of each of the PPINs and IPN matrices were compared with each other. If the corresponding entities in the two matrices were equal, the two clustering methods resulted in the same clusters. The following four conditions occurred: Agreements; n11 (Number of paired entities in the similarity matrices in which both are 1) and n00 (Number of paired entities in the similarity matrices in which both are 0), Disagreements; n10 (Number of paired entities that are 1 in the PPIN similarity matrix and 0 in the IPN similarity matrix) and n01 (Number of paired entities that are 1 in the IPN similarity matrix and 0 in the PPIN similarity matrix).

There are several benchmarking indices to measure the degree of agreement and disagreement between the two matrices [55]. Some of the indices used in this study are as follows:

$$ \begin{array}{c}\hfill Rand=\frac{\left({n}_{11}+{n}_{00}\right)}{\left({n}_{11}+{n}_{10}+{n}_{01}+{n}_{00}\right)}\hfill \\ {}\hfill Jaccard=\frac{n_{11}}{\left({n}_{11}+{n}_{10}+{n}_{01}\right)}\hfill \\ {}\hfill Minkowski=\sqrt{\frac{\left({n}_{10}+{n}_{01}\right)}{\left({n}_{11}+{n}_{01}\right)}}\hfill \\ {}\hfill \mathrm{Folkes}-\mathrm{Mallows}=\frac{n_{11}}{\sqrt{\left({n}_{11}+{n}_{01}\right)\times \left({n}_{11}+{n}_{10}\right)}}\hfill \end{array} $$

In order to perform biological evaluation of IPN modules, Enrichr software was used [56]. In this web-based tool, significantly enriched terms are extracted based on the Gene Ontology, Biological Processes [38], Kegg Orthology [57] and Reactome databases [58]. The combined score; consisting of the Z-score and adjusted *p*-value, was used to rank and define enriched terms. This validation was done for the modules defined by MCL algorithm as a superior algorithm in our comparison.

## Additional files

Additional file 1:
**IPN.** The IPN derived from the four different PPINs of four species is shown. They include human, worm, fruit fly, and rat PPINs from down left clockwise. Nodes with greater degrees are indicated as circles with larger diameters and each module is shown with the specific node color. (JPEG 4673 kb)
Additional file 2:
**Network Parameters.** Some network parameter are included to gain clear insight about four PPINs and IPN. Also, all calculated external clustering measures and indices are presented in this table. The ranges for Rand, Jaccard and Fowlkes-Mallows are [0, 1] which are presented in percent whereas the Minkowski rang is [0, +∞). These values are computed for all species and five clustering algorithms. (DOCX 17 kb)
Additional file 3:
**Biological evidence.** In this file, the IPN nodes, the MCL modules, the human proteins in OPSs and the enriched terms of proteins consist IPN modules are presented. The enrichment analysis of each module presented separately in different sheets. The human proteins were used to perform this analysis. (XLSX 38 kb)
Additional file 4:
**IPN reconstruction steps.** First, the mitochondrial proteins are extracted from the *UniProt* database, and then the reviewed proteins are filtered. Using the Needleman and Wunsch algorithm, the homologous proteins are identified in the OPSs. In the next step, four distinct PPINs from four species are identified from the STRING database. Finally, the IPN is created by finding the interlog proteins in all four PPINs. In each step some proteins are pretermitted to discern conserved structures. (PDF 123 kb)
Additional file 5:
**Some details regarding the five clustering algorithm algorithms (MCL, RNSC, CR, LD and GAPPI) are described briefly.** (DOCX 13 kb)

## References

[1] Barabási AL (2011). The network takeover. Nat Phys.

[2] Carvunis AR (2011). From proteins and their interactions to evolutionary principles of biological systems. Université Joseph Fourier.

[3] Hou J, Chi X (2012). Predicting protein functions from PPI networks using functional aggregation. Math Biosci.

[4] Srivas R, Hannum G, Ruscheinski J, Ono K, Wang P-L, Smoot M (2011). Assembling global maps of cellular function through integrative analysis of physical and genetic networks. Nat Protoc.

[5] Bandyopadhyay S, Kelley R, Krogan NJ, Ideker T (2008). Functional maps of protein complexes from quantitative genetic interaction data. PLoS Comput Biol.

[6] Sharan R, Ulitsky I, Shamir R (2007). Network-based prediction of protein function. Mol Syst Biol.

[7] Sharan R, Ideker T (2006). Modeling cellular machinery through biological network comparison. Nat Biotechnol.

[8] Futschik ME, Chaurasia G, Herzel H (2007). Comparison of human protein–protein interaction maps. Bioinformatics.

[9] Luonan C, Rui-Sheng W, Xiang-Sun Z (2009). Biomolecular networks methods and applications in systems biology.

[10] Braun P, Tasan M, Dreze M, Barrios-Rodiles M, Lemmens I, Yu H (2009). An experimentally derived confidence score for binary protein-protein interactions. Nat Methods.

[11] Ngounou Wetie AG, Sokolowska I, Woods AG, Roy U, Deinhardt K, Darie CC (2013). Protein-protein interactions: switch from classical methods to proteomics and bioinformatics-based approaches. Cellular and Molecular Life Sciences: CMLS.

[12] Junker BH, Schreiber F (2007). Analysis of biological networks.

[13] Yu D, Kim M, Xiao G, Hwang TH (2013). Review of biological network data and its applications. Genomics Inform.

[14] Vespignani A (2003). Evolution thinks modular. Nat Genet.

[15] Hartwell LH, Hopfield JJ, Leibler S, Murray AW (1999). From molecular to modular cell biology. Nature.

[16] Pizzuti C, Rombo SE (2014). Algorithms and tools for protein-protein interaction networks clustering, with a special focus on population-based stochastic methods. Bioinformatics.

[17] Blatt M, Wiseman S, Domany E (1996). Superparamagnetic clustering of data. Phys Rev Lett.

[18] Hartuv E, Shamir R (2000). A clustering algorithm based on graph connectivity. Inf Process Lett.

[19] Enright AJ, Van Dongen S, Ouzounis CA (2002). An efficient algorithm for large-scale detection of protein families. Nucleic Acids Res.

[20] King AD, Przulj N, Jurisica I (2004). Protein complex prediction via cost-based clustering. Bioinformatics.

[21] Arnau V, Mars S, Marín I (2005). Iterative cluster analysis of protein interaction data. Bioinformatics.

[22] Vazquez A, Flammini A, Maritan A, Vespignani A (2003). Global protein function prediction from protein-protein interaction networks. Nat Biotechnol.

[23] Altaf-Ul-Amin M, Shinbo Y, Mihara K, Kurokawa K, Kanaya S (2006). Development and implementation of an algorithm for detection of protein complexes in large interaction networks. BMC Bioinformatics.

[24] Rives AW, Galitski T (2003). Modular organization of cellular networks. Proc Natl Acad Sci U S A.

[25] Maciag K, Altschuler SJ, Slack MD, Krogan NJ, Emili A, Greenblatt JF (2006). Systems-level analyses identify extensive coupling among gene expression machines. Mol Syst Biol.

[26] Samanta MP, Liang S (2003). Predicting protein functions from redundancies in large-scale protein interaction networks. Proc Natl Acad Sci U S A.

[27] Zhou H (2003). Network landscape from a Brownian particle’s perspective. Phys Rev E.

[28] Bader GD, Betel D, Hogue CWV (2003). Bind: the biomolecular interaction network database. Nucleic Acids Res.

[29] Spirin V, Mirny LA (2003). Protein complexes and functional modules in molecular networks. Proc Natl Acad Sci U S A.

[30] Dunn R, Dudbridge F, Sanderson CM (2005). The use of edge-betweenness clustering to investigate biological function in protein interaction networks. BMC Bioinformatics.

[31] Pereira-Leal JB, Enright AJ, Ouzounis CA (2004). Detection of functional modules from protein interaction networks. Proteins.

[32] Brohée S, van Helden J (2006). Evaluation of clustering algorithms for protein-protein interaction networks. BMC Bioinformatics.

[33] Mewes HW, Ruepp A, Theis F, Rattei T, Walter M, Frishman D (2011). MIPS: curated databases and comprehensive secondary data resources in 2010. Nucleic Acids Res.

[34] Mewes HW, Frishman D, Güldener U, Mannhaupt G, Mayer K, Mokrejs M (2002). MIPS: a database for genomes and protein sequences. Nucleic Acids Res.

[35] Mewes HW, Dietmann S, Frishman D, Gregory R, Mannhaupt G, Mayer KFX (2008). MIPS: analysis and annotation of genome information in 2007. Nucleic Acids Res.

[36] Wang Z, Zhang J (2007). In search of the biological significance of modular structures in protein networks. PLoS Comput Biol.

[37] Phan HTT, Sternberg MJE (2012). PINALOG: a novel approach to align protein interaction networks–implications for complex detection and function prediction. Bioinformatics.

[38] Ashburner M, Ball CA, Blake JA, Botstein D, Butler H, Cherry JM (2000). Gene ontology: tool for the unification of biology. Gene.

[39] Sevilla JL, Segura V, Podhorski A, Guruceaga E, Mato JM, Martínez-Cruz LA (2005). Correlation between gene expression and GO semantic similarity. IEEE ACM Trans Comput Biol Bioinformatics.

[40] Lord PW, Stevens RD, Brass A, Goble CA (2003). Investigating semantic similarity measures across the Gene Ontology: the relationship between sequence and annotation. Bioinformatics.

[41] du Plessis L, Skunca N, Dessimoz C (2011). The what, where, how and why of gene ontology–a primer for bioinformaticians. Brief Bioinform.

[42] Cho YR, Hwang W, Ramanathan M, Zhang A (2007). Semantic integration to identify overlapping functional modules in protein interaction networks. BMC Bioinformatics.

[43] Dotan-Cohen D, Letovsky S, Melkman AA, Kasif S (2009). Biological process linkage networks. PLoS One.

[44] Luciani D, Bazzoni G (2012). From networks of protein interactions to networks of functional dependencies. BMC Syst Biol.

[45] Khatri P, Drăghici S (2005). Ontological analysis of gene expression data: current tools, limitations, and open problems. Bioinformatics.

[46] Jafari M, Sadeghi M, Mirzaie M, Marashi SA, Rezaei-Tavirani M (2013). Evolutionary conserved motifs and modules in mitochondrial protein interaction network. Mitochondrion.

[47] Guimerà R, Nunes Amaral LA (2005). Functional cartography of complex metabolic networks. Nature.

[48] Blondel VD, Guillaume JL, Lambiotte R, Lefebvre E (2008). Fast unfolding of communities in large networks. J Stat Mech Theor Exp.

[49] Lambiotte R, Ausloos M, Hołyst JA (2007). Majority model on a network with communities. Phys Rev E.

[50] Jafari M, Primo V, Smejkal GB, Moskovets EV, Kuo WP, Ivanov AR (2012). Comparison of in-gel protein separation techniques commonly used for fractionation in mass spectrometry-based proteomic profiling. Electrophoresis.

[51] Chen L, Wang RS, Zhang XS (2009). Biomolecular networks: methods and applications in systems biology: Wiley.

[52] Zhang A (2009). Protein interaction networks: computational analysis: Cambridge University Press.

[53] Jansen R, Yu H, Greenbaum D, Kluger Y, Krogan NJ, Chung S (2003). A Bayesian networks approach for predicting protein-protein interactions from genomic data. Science.

[54] Jiang D, Tang C, Zhang A (2004). Cluster analysis for gene expression data: a survey. IEEE Trans Knowl Data Eng.

[55] Campello RJGB (2007). A fuzzy extension of the Rand index and other related indexes for clustering and classification assessment. Pattern Recogn Lett.

[56] Chen EY, Tan CM, Kou Y, Duan Q, Wang Z, Meirelles GV (2013). Enrichr: interactive and collaborative HTML5 gene list enrichment analysis tool.

[57] Kanehisa M, Goto S (2000). KEGG: kyoto encyclopedia of genes and genomes. Nucleic Acids Res.

[58] Croft D, Mundo AF, Haw R, Milacic M, Weiser J, Wu G (2014). The Reactome pathway knowledgebase. Nucleic Acids Res.

[59] Tieri P, Nardini C (2013). Signalling pathway database usability: lessons learned. Mol BioSyst.

[60] von Mering C, Jensen LJ, Snel B, Hooper SD, Krupp M, Foglierini M (2005). STRING: known and predicted protein-protein associations, integrated and transferred across organisms. Nucleic Acids Res.

[61] Szklarczyk D, Franceschini A, Kuhn M, Simonovic M, Roth A, Minguez P (2011). The STRING database in 2011: functional interaction networks of proteins, globally integrated and scored. Nucleic Acids Res.

[62] Dobzhansky T (1973). Nothing in biology makes sense except in the light of evolution. Am Biol Teach.

[63] Wu CH, Apweiler R, Bairoch A, Natale DA, Barker WC, Boeckmann B (2006). The Universal Protein Resource (UniProt): an expanding universe of protein information. Nucleic Acids Res.

