# Supplementary figures and images for: Interlog protein network: an evolutionary benchmark of protein interaction networks for the evaluation of clustering algorithms
Source: BMC Bioinformatics. 2015 Oct 5;16:319. doi: 10.1186/s12859-015-0755-1 (PMC4595048; doi:10.1186/s12859-015-0755-1)

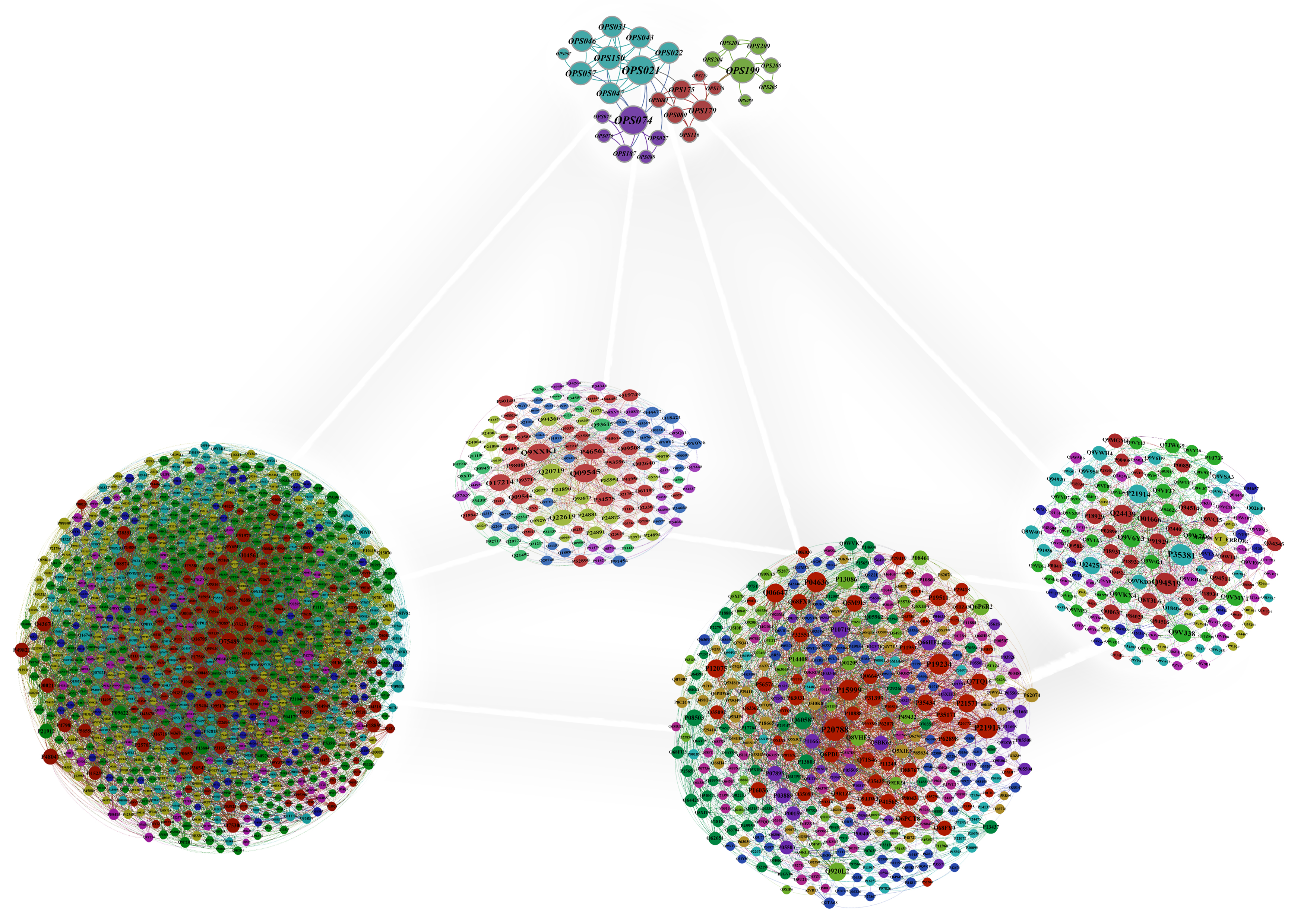

Supplement: Additional file 1: — IPN. The IPN derived from the four different PPINs of four species is shown. They include human, worm, fruit fly, and rat PPINs from down left clockwise. Nodes with greater degrees are indicated as circles with larger diameters and each module is shown with the specific node color. (JPEG 4673 kb) [file 12859_2015_755_MOESM1_ESM.jpg]
