# Supplementary material for: Interlog protein network: an evolutionary benchmark of protein interaction networks for the evaluation of clustering algorithms
Source: BMC Bioinformatics. 2015 Oct 5;16:319. doi: 10.1186/s12859-015-0755-1 (PMC4595048; doi:10.1186/s12859-015-0755-1)
Supplement: Additional file 2: — Network Parameters. Some network parameter are included to gain clear insight about four PPINs and IPN. Also, all calculated external clustering measures and indices are presented in this table. The ranges for Rand, Jaccard and Fowlkes-Mallows are [0, 1] which are presented in percent whereas the Minkowski rang is [0, +∞). These values are computed for all species and five clustering algorithms. (DOCX 17 kb) [file 12859_2015_755_MOESM2_ESM.docx]

**Additional file 2:** Some network parameter are included to gain clear insight about four PPINs and IPN. Also, all calculated external clustering measures and indices are presented in this table. The ranges for Rand, Jaccard and Fowlkes-Mallows are [0,1] which are presented in percent whereas the Minkowski rang is [0,+∞). These values are computed for all species and five clustering algorithms.

| Dataset | No. nodes | No. edges | Mean degree | Mean clust. coeff. | Network diameter | Graph density | Indices | MCL | RNSC | LD | CR | GAPPI |
| --- | --- | --- | --- | --- | --- | --- | --- | --- | --- | --- | --- | --- |
| IPN | **29** | **63** | **4.345** | **0.625** | **6** | **0.155** |  |  |  |  |  |  |
| Human PPIN | 858 | 8745 | 20.385 | 0.354 | 8 | 0.024 | Rand | 77% | 86% | 75% | 74% | 84% |
|  |  |  |  |  |  |  | Jaccard | 50% | 45% | 35% | 35% | 49% |
|  |  |  |  |  |  |  | Fowlkes-Mallows | 68% | 64% | 52% | 51% | 67% |
|  |  |  |  |  |  |  | Minkowski | 0.736 | 0.975 | 0.994 | 0.994 | 0.779 |
|  |  |  |  |  |  |  |  |  |  |  |  |  |
| Rat PPIN | 391 | 2431 | 12.435 | 0.392 | 10 | 0.032 | Rand | 90% | 75% | 75% | 73% | 78% |
|  |  |  |  |  |  |  | Jaccard | 77% | 35% | 33% | 33% | 45% |
|  |  |  |  |  |  |  | Fowlkes-Mallows | 87% | 56% | 49% | 50% | 63% |
|  |  |  |  |  |  |  | Minkowski | 0.500 | 1.267 | 0.992 | 1.038 | 0.941 |
|  |  |  |  |  |  |  |  |  |  |  |  |  |
| Fruit Fly PPIN | 159 | 676 | 8.503 | 0.538 | 9 | 0.054 | Rand | 87% | 84% | 75% | 70% | 79% |
|  |  |  |  |  |  |  | Jaccard | 74% | 40% | 33% | 23% | 46% |
|  |  |  |  |  |  |  | Fowlkes-Mallows | 85% | 57% | 49% | 37% | 63% |
|  |  |  |  |  |  |  | Minkowski | 0.541 | 0.971 | 0.957 | 1.078 | 0.873 |
|  |  |  |  |  |  |  |  |  |  |  |  |  |
| Worm PPIN | 133 | 618 | 9.293 | 0.485 | 7 | 0.07 | Rand | 98% | 77% | 77% | 77% | 85% |
|  |  |  |  |  |  |  | Jaccard | 95% | 41% | 43% | 45% | 61% |
|  |  |  |  |  |  |  | Fowlkes-Mallows | 97% | 64% | 60% | 63% | 76% |
|  |  |  |  |  |  |  | Minkowski | 0.227 | 1.195 | 0.911 | 0.931 | 0.742 |
