# Supplementary material for: Interlog protein network: an evolutionary benchmark of protein interaction networks for the evaluation of clustering algorithms
Source: BMC Bioinformatics. 2015 Oct 5;16:319. doi: 10.1186/s12859-015-0755-1 (PMC4595048; doi:10.1186/s12859-015-0755-1)
Supplement: Additional file 4: — IPN reconstruction steps. First, the mitochondrial proteins are extracted from the UniProt database, and then the reviewed proteins are filtered. Using the Needleman and Wunsch algorithm, the homologous proteins are identified in the OPSs. In the next step, four distinct PPINs from four species are identified from the STRING database. Finally, the IPN is created by finding the interlog proteins in all four PPINs. In each step some proteins are pretermitted to discern conserved structures. (PDF 123 kb) [file 12859_2015_755_MOESM4_ESM.pdf]

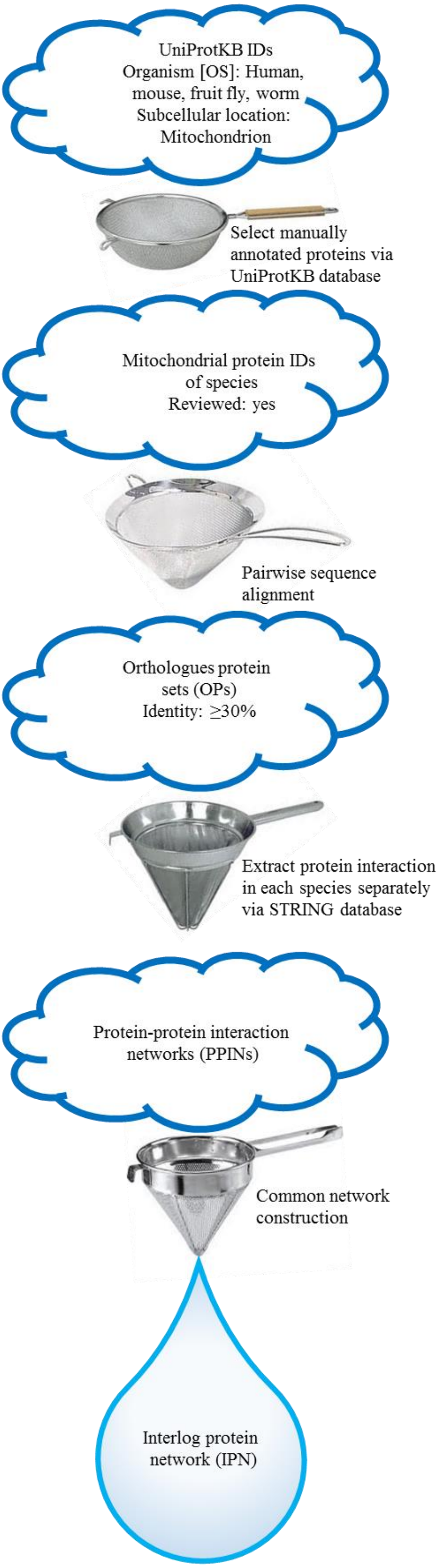
UniProtKB IDs
Organism [OS]: Human, mouse, fruit fly, worm
Subcellular location: Mitochondrion
Select manually annotated proteins via UniProtKB database
Mitochondrial protein IDs of species
Reviewed: yes
Pairwise sequence alignment
Orthologues protein sets (OPs)
Identity: ≥30%
Extract protein interaction in each species separately via STRING database
Protein-protein interaction networks (PPINs)
Common network construction
Interlog protein network (IPN)
