# Supplementary material for: Interlog protein network: an evolutionary benchmark of protein interaction networks for the evaluation of clustering algorithms
Source: BMC Bioinformatics. 2015 Oct 5;16:319. doi: 10.1186/s12859-015-0755-1 (PMC4595048; doi:10.1186/s12859-015-0755-1)
Supplement: Additional file 5: — Some details regarding the five clustering algorithm algorithms (MCL, RNSC, CR, LD and GAPPI) are described briefly. (DOCX 13 kb) [file 12859_2015_755_MOESM5_ESM.docx]

**Additional file 5.** Some details about the five clustering algorithm algorithms (Markov chain clustering (MCL), Restricted Neighborhood Search Clustering (RNSC), cartographic representation (CR), Laplacian dynamics (LD) and Genetic Algorithm to find communities in Protein-Protein Interaction networks (GAPPI)) are described briefly.

The first method is MCL which uses Markov chain and simulation of the flow on graph. In each iteration, an "inflation" is used to distinguish between weak and strong flow areas. This process leads to split the graph into regions with high-flow and no-flow boundaries. The number of clusters is determined by the value of inflation.

Next method is RNSC which works by a cost function which is defined based on the number of edges within and between clusters. At the first step, random clusters are chosen, then by replacing the nodes in different clusters, the algorithm tries to reduce cost. The optimum values of parameters are according to prior study.

In the third method, “cartographic representation" (CR) is used to identify clusters. This method optimizes objective function by simulated annealing algorithm which applies stochastic technique to perform an exhaustive search for the local optimum parts. In the other words, it explores low-cost configuration with no entrapment in local high-cost region. The most important parameter called computational temperature T is defined as the cluster number.

The forth module finding algorithm is a greedy algorithm, available under Gephi program. The Laplacian dynamics (LD) is used to fast unfolding communities or module by this algorithms. This algorithm works in a very short time and with no network-size limitations. The resolution power of this method identifies module numbers.

The final module finding algorithm is a class of adaptive general-purpose search techniques inspired by natural evolution. The genetic algorithm to find communities in Protein-Protein Interaction networks called GAPPI use a fitness function based on network topology. This algorithm is available under MATLAB code following this address http://staff.icar.cnr.it/pizzuti/codes.html.
